# Gut microbiota mediates the beneficial effects of exercise on autism-like behaviors

**DOI:** 10.1186/s12866-025-04632-x

**Published:** 2026-01-12

**Authors:** Jiugen Zhong, Baoyuan Zhu, Zhi Zou, Yinhua Li, Yanqing Feng, Kai Wu, Xiaohui Hou

**Affiliations:** 1 Guangdong Provincial Key Laboratory of Physical Activity and Health Promotion, Guangzhou, Guangdong Province 500510 China; 2 Medical School of Shaoxing University, Shaoxing, Zhejiang Province 312000 China; 3 Guangzhou Sport University, Guangzhou, Guangdong Province 500510 China; 4 Guangdong Key Laboratory of Human Sports Performance Science, Guangzhou, Guangdong Province 500510 China; 5 School of Biomedical Sciences and Engineering, South China University of Technology, Guangzhou International Campus, Guangzhou, Guangdong Province 511442 China; 6 National Engineering Research Center for Tissue Restoration and Reconstruction, South China University of Technology, Guangzhou, Guangdong Province 510006 China; 7 Department of Nuclear Medicine and Radiology, Institute of Development, Aging and Cancer, Tohoku University, Sendai, 980-8575 Japan; 8 School of Material Science and Engineering, South China University of Technology, Guangzhou, 510006 China

**Keywords:** Gut microbiota, Exercise intervention, Autism spectrum disorder, Fecal microbiota transplantation, Short-chain fatty acids, Neurotransmitters

## Abstract

**Background:**

The gut-brain axis plays a critical role in autism spectrum disorder (ASD), but the mechanisms through which exercise modulates gut microbiota, short-chain fatty acids (SCFAs), and central neurotransmitters to ameliorate ASD-like behaviors remain unclear. This study aimed to investigate the effects of exercise on ASD-like behaviors, gut microbiota, and metabolism in a valproic acid (VPA)-induced ASD rat model and to validate these findings via fecal microbiota transplantation (FMT).

**Methods:**

ASD rat models were established through prenatal exposure to VPA and divided into four groups: exercise (E_ASD), non-exercise (ASD), FMT, and sham FMT (sFMT). The E_ASD group underwent 6 weeks of voluntary wheel running, while the FMT group received fecal microbiota from the E_ASD group for 4 weeks. Behavioral assessments were conducted to evaluate cognitive and social functions. Fecal microbiota composition was analyzed via 16S rRNA sequencing, while SCFAs and neurotransmitters were measured using gas and liquid chromatography-mass spectrometry.

**Results:**

Six weeks of voluntary exercise significantly alleviated ASD-like behaviors, particularly improving social interactions. Exercise also altered gut microbiota composition, increasing *Limosilactobacillus* and *Lactobacillus* while decreasing *Allobaculum*. Additionally, SCFAs and neurotransmitter levels in the prefrontal cortex were modulated. Notably, FMT from the exercise group replicated these behavioral and metabolic improvements in ASD rats. Exercise improves ASD-like behaviors by modulating gut microbiota, SCFAs, and neurotransmitter levels, and FMT offers further validation of these effects.

**Conclusion:**

These findings highlight exercise and FMT as promising strategies for alleviating ASD-related symptoms through gut-brain axis modulation.

**Supplementary Information:**

The online version contains supplementary material available at 10.1186/s12866-025-04632-x.

## Background

Autism spectrum disorder (ASD) is a serious neurodevelopmental disorder characterized by impaired social communication as well as the occurrence of narrow interests and repetitive behaviors [1]. According to data from the Centers for Disease Control and Prevention Morbidity and Mortality Weekly Report, the overall ASD incidence is one in thirty-six children aged 8 years in the USA [2]. The pathology of ASD remains unclear, and there is no specific treatment available. Therefore, finding effective ways to improve and alleviate the core symptoms of ASD has become a focal point in autism rehabilitation research. In recent years, interest in the relationship between the gut microbiota and human health has increased, providing new perspectives for understanding the pathogenesis of various diseases [3].

Accumulating evidence indicates that the gut microbiota plays a significant role in the onset and progression of ASD in both humans and animals [4–8]. Studies have also shown that modulating the gut microbiota through interventions such as probiotic supplementation and fecal microbiota transplantation (FMT) can alleviate symptoms in individuals with ASD [9, 10]. The bidirectional communication between the gut microbiota and the brain involves multiple pathways, primarily the immune system, neuroendocrine pathways, metabolism, and the vagal nerve pathway [11–13]. As an endocrine-like organ, the microbiota not only produces many metabolic products, such as tryptophan and short-chain fatty acids (SCFAs), which are involved in energy balance and metabolism but also synthesizes and releases several neurotransmitters, similar to those in the brain [14]. Moreover, neurotransmitters are the most important intermediaries in the communication of neurons, and dysfunction of neurotransmitters may explain the mechanism of excitatory or inhibitory imbalance in ASD patients. Therefore, regulating the microbiota and its metabolism, as well as microbiota transplantation, has consistently been regarded as a potential therapeutic target and intervention strategy for ASD [11, 15].

Furthermore, research has shown that the microbiota can be influenced by exercise, as can antibiotics, probiotics, nutrients, and microbiota transplantation. Additionally, research indicates that exercise can improve symptoms related to various diseases, including colitis, diabetes, and Alzheimer’s disease, by altering the gut microbiota [16–19]. In addition, extensive studies have demonstrated the positive effects of exercise on the core symptoms of ASD [20–27], as well as improvements in quality of life [24], but the underlying mechanism of the ameliorative effects of exercise on ASD remains unclear. Moreover, the regulatory effects of exercise on central neurotransmitters have also been confirmed by numerous studies [28–31]. However, there is currently a lack of evidence regarding whether the ameliorating effects of exercise on ASD are related to alterations in the microbiota and their connection to neurotransmitter levels.

In this study, we successfully established a rat model of ASD through prenatal exposure to valproic acid (VPA). The rats were then randomly divided into four groups: the ASD group, the six-week voluntary wheel-running exercise intervention (E-ASD) group, the FMT group, in which the feces of the rats in the E-ASD group were transplanted, and the saline sham transplant (sFMT) group. This study reveals how exercise modulates the gut microbiota to influence metabolic changes and ameliorate autism-like behaviors by comparing behavioral performance, gut microbiota composition, SCFAs, and neurotransmitter changes in rats receiving exercise interventions and FMT. These findings highlight the potential of exercise interventions as a therapeutic strategy for modulating ASD and offer new perspectives on the use of microbiota transplantation as a treatment approach.

## Methods

### Animals and housing conditions

Pregnant Sprague–Dawley rats were obtained from the Laboratory Animal Center of Southern Medical University (P. R. China). All experimental procedures were approved by the Ethics Committee of Guangzhou Sport University and were conducted in accordance with the National Institutes of Health Guide for the Care and Use of Laboratory Animals (NIH Publication No. 85–23). On gestational day 12.5, the pregnant rats received an intraperitoneal injection of either 400 mg/kg valproic acid sodium salt (VPA; P4543, Sigma, USA) to induce autism-like phenotypes in the offspring, or an equivalent volume of normal saline as a control, as previously described [32]. The pregnant rats were housed individually and provided with ad libitum access to food and water. They were allowed to rear their litters without intervention, except for those designated for growth and behavioral development assessments. Offspring were weaned on postnatal day 23. All groups were maintained under identical housing conditions to minimize any potential variability in social behaviors caused by differences in housing environments. The light/dark cycle was carefully controlled and set to 12 h of light and 12 h of darkness each day to simulate a natural diurnal rhythm, and the ambient temperature in the housing area was consistently maintained at 23 °C ± 2 °C to ensure a stable and comfortable environment for the animals. The gut microbiota composition, SCFAs, and neurotransmitter levels of all rats were assessed both before and after the intervention, along with behavioral testing. The methods used for behavioral testing (including the open field test and three-chamber social interaction test), 16S rRNA gene sequencing, SCFA analysis, and neurotransmitter analysis were consistent with those described in previous publications [33]. For more detailed descriptions of the experimental protocols and analytical methods, please refer to the Supplementary Materials.

### Exercise intervention protocol

Rats in the E_ASD group were provided with a voluntary running wheel (35 cm diameter) to encourage physical activity. The intervention began on postnatal day 28 (P28) and lasted 42 days. During this period, the rats were allowed to use the running wheel continuously in their home cages and their running distances were automatically recorded by a computerized system. The system tracked the total running distance of each rat on a daily basis to accurately monitor their physical activity levels during the intervention. Running distance was calculated by multiplying the total number of wheel revolutions by the circumference of the 35-cm wheel. Daily running distance was then derived over each 24-hour cycle, providing a high-precision, high-resolution measure of voluntary activity while minimizing disturbance to the animals. The resulting running-distance data were further used as a quantitative indicator of exercise intensity and were incorporated as a covariate in the behavioral statistical analyses.

### FMT procedure

To investigate whether the increase in behavioral performance is due to changes in the gut microbiome, fecal samples from the rats in the exercise group were transplanted into the rats in the non-exercise group. This was accomplished via gavage starting in the third week of the exercise intervention. The method of preparation followed that of Sampson et al. [34]. Fresh fecal pellets from exercised group rats were collected at 9:00 a.m. and diluted in sterile phosphate-buffered saline (PBS) at a 1:5 ratio. The mixture was thoroughly stirred to form a uniform suspension. Next, the suspension was passed through a 0.5 mm mesh filter to remove food debris, yielding a secondary suspension. This was then centrifuged at 6000 rpm for 15 min at 4 °C. After discarding the supernatant, the resulting pellet was resuspended in the same volume of sterile PBS used initially, generating the final fecal bacterial filtrate. This suspension was administered to recipient rats by oral gavage at a dosage of 1 mL per 100 g of body weight, once daily for 4 weeks.

### Bioinformatics analysis

The raw 16S rRNA gene sequencing data were initially processed via the standard QIIME2 pipeline, which included quality filtering, adapter trimming, and chimera removal to ensure the overall accuracy and reliability of the dataset. The DADA2 algorithm was subsequently applied for sequence dereplication and quality control, resulting in the generation of amplicon sequence variants with 100% sequence similarity. Low-abundance taxa present in less than 10% of the samples were filtered out to ensure data reliability. The microbial composition was visualized by selecting the top ten most abundant genera. Alpha diversity indices, including Shannon, Simpson, Chao1, and ACE indices, were calculated at the genus level, and intergroup differences in within-sample diversity were assessed using the Wilcoxon rank-sum test. Beta diversity was analyzed using principal coordinate analysis (PCoA) based on the Bray-Curtis distance matrix, and intergroup differences were calculated using permutational multivariate analysis of variance (PERMANOVA) with 999 permutations. Differentially abundant taxa between groups were identified via linear discriminant analysis effect size (LEfSe), with statistical significance determined via Kruskal‒Wallis and Wilcoxon tests (*p* < 0.05) and a linear discriminant analysis (LDA) score threshold set at > 4. To investigate the potential interactions among gut bacterial taxa, SCFAs, and neurotransmitters, a correlation-based network analysis was performed. Pairwise Spearman correlation coefficients were calculated based on the relative abundance or concentration levels of significantly different bacterial genera, SCFAs, and neurotransmitters. Only statistically significant correlations with FDR-corrected *p* values < 0.05 and absolute correlation coefficients |r| ≥ 0.3 were retained for network construction. The resulting correlation network was visualized using Cytoscape software (version 3.9.1). In the network, each node represents a bacterial genus, SCFA, or neurotransmitter, while edges indicate significant correlations between these variables. The direction and strength of the correlations are denoted by the color and thickness of the edges: red edges represent positive correlations, whereas blue edges indicate negative correlations.

### Statistical analysis

All statistical analyses were performed using SPSS software (version 19.0, IBM Corp., Armonk, NY, USA), GraphPad Prism (version 8.0, GraphPad Software Inc., San Diego, CA, USA), and R software (version 4.3.2). Prior to statistical testing, data normality was assessed using the Shapiro-Wilk test, and homogeneity of variance was evaluated using Levene’s test. For normally distributed data with equal variances, independent t-tests were used for comparisons between two groups. For data that did not meet the assumptions of parametric tests, the Mann-Whitney U test was applied for comparison. For comparisons among the three groups, one-way ANOVA followed by Tukey’s post hoc test was performed when the data met the assumptions of normality and homogeneity of variance; when these assumptions were not satisfied, the Kruskal–Wallis test followed by Dunn’s multiple comparison correction was applied. Spearman’s rank correlation analysis was conducted to identify significant associations among differentially abundant bacterial genera, SCFAs, and neurotransmitters. To reduce the risk of type I errors due to multiple comparisons, the Benjamini–Hochberg procedure was applied to control the false discovery rate (FDR), with statistical significance set at *p* < 0.05. All statistical results were visualized using a range of analytical tools: box plots and violin plots were generated using GraphPad Prism (v9.0); correlation heatmaps and network analyses were carried out with R packages including ggplot2, pheatmap, and igraph. Microbial co-occurrence networks and metabolite interaction networks were further refined and visualized using Cytoscape software (v3.9.1).

## Results

### Voluntary wheel running alleviates autism-like behaviors

**Fig. 1:**
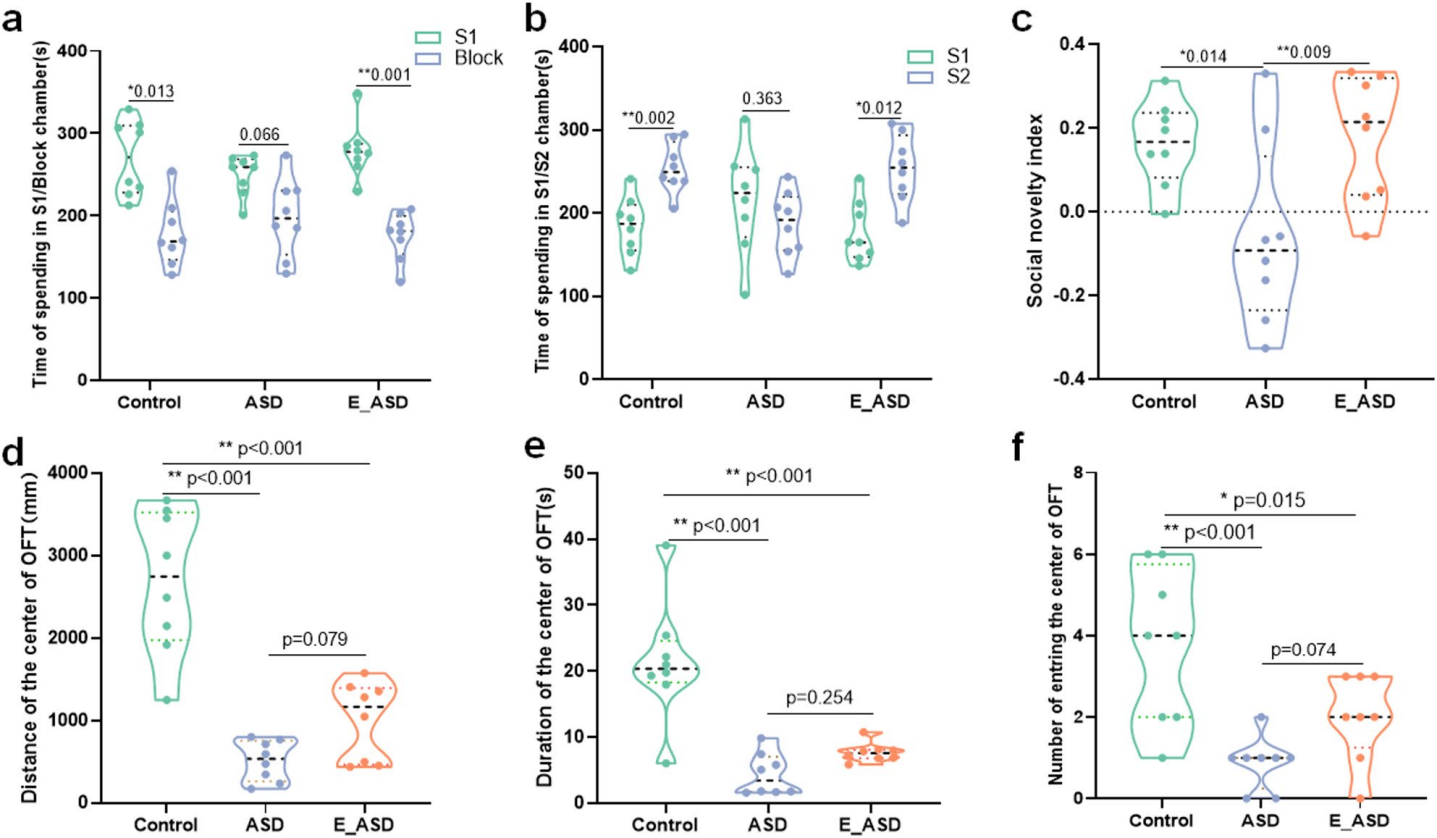
Behavioral assessments across three groups: control, ASD, and E_ASD. **a**. Time spent in the stranger-1 (S1) chamber versus the empty chamber (Block) during the sociability phase. **b**. Time spent in the stranger-1 (S1) versus stranger-2 (S2) chamber during the social novelty preference phase. **c**. Social novelty index calculated as (S2 − S1) / (S2 + S1). d. Distance from the center during the open field test (OFT), reflecting anxiety-like behavior. e. Duration of time spent in the center of the OFT. f. Number of entries into the center of the OFT. *p < 0.05, **p < 0.01 indicate significant differences between groups. *N* = 8 (male = 4, female = 4)

In our previous study, we measured behavioral performance, gut microbiota composition, SCFAs, and neurotransmitter levels prior to the intervention, thereby confirming the successful establishment of the ASD-like rat model [33]. To assess whether exercise could ameliorate autism-like behaviors in VPA rats, voluntary wheel-running was initiated at postweaning (P28) and continued for six weeks (Fig. S1a). Throughout the intervention, running distance increased steadily across individuals, indicating sustained and regular engagement in voluntary exercise (Fig. S2). In the three-chamber social test, during the sociability phase, both the control and E_ASD groups spent significantly more time in the stranger-1 (S1) chamber than in their respective empty chambers (Block), whereas the ASD group showed no clear preference between S1 and Block (Fig. 1a). This pattern indicates an impairment in basic social interest in the ASD group, while early voluntary wheel-running partially restored this preference for conspecific interaction. During the social novelty preference phase, the control and E_ASD groups spent more time in the stranger-2 (S2) chamber than in their corresponding S1 chambers, whereas the ASD group displayed no difference between S1 and S2 (Fig. 1b). Consistently, the social novelty index in the ASD group was significantly lower than that in both the control and E_ASD groups, whereas no significant difference was observed between the control and E_ASD groups (Fig. 1c), suggesting that exercise intervention partially ameliorated the VPA-induced deficit in social novelty. Although no significant differences in the overall sociability index were observed among the three groups (Fig. S3a), the E_ASD group showed an improving trend compared with the ASD group. In the open-field test (OFT), the ASD group exhibited more pronounced anxiety-like behavior, reflected by a greater average distance from the center, shorter time spent in the central area, and fewer center entries relative to the control group (Fig. 1d–f), whereas the E_ASD group showed partial improvement but did not completely return to control levels. Meanwhile, no significant differences were found among the groups in total distance traveled, average locomotor speed, or central-zone movement speed (Fig. S3b–d), indicating that the intervention did not fully restore the initial behavioral state and that longer-term exercise or multimodal therapeutic approaches may be required for more complete recovery.

### Early voluntary wheel running alters the gut microbiota, SCFAs, and neurotransmitters in ASD rats

**Fig. 2:**
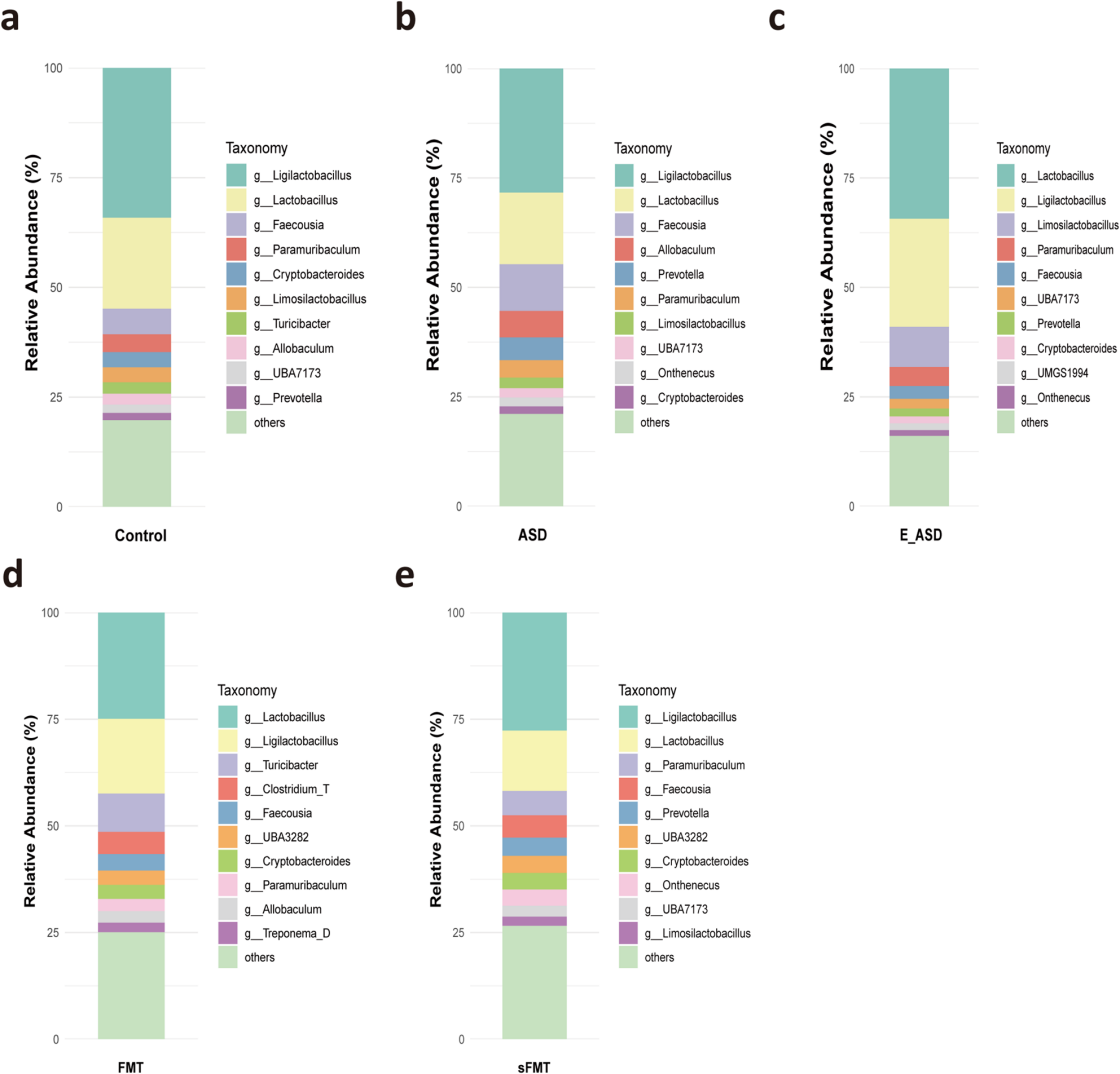
Comparative composition of dominant gut microbial genera across groups. **a**. Relative abundance of gut microbiota at the genus level in the Control group. **b**. Relative abundance of gut microbiota at the genus level in the ASD group. **c**. Relative abundance of gut microbiota at the genus level in the E_ASD group. **d**. Relative abundance of gut microbiota at the genus level in the FMT group. **e**. Relative abundance of gut microbiota at the genus level in the sFMT group. N =8 (male = 4, female = 4)

**Fig. 3:**
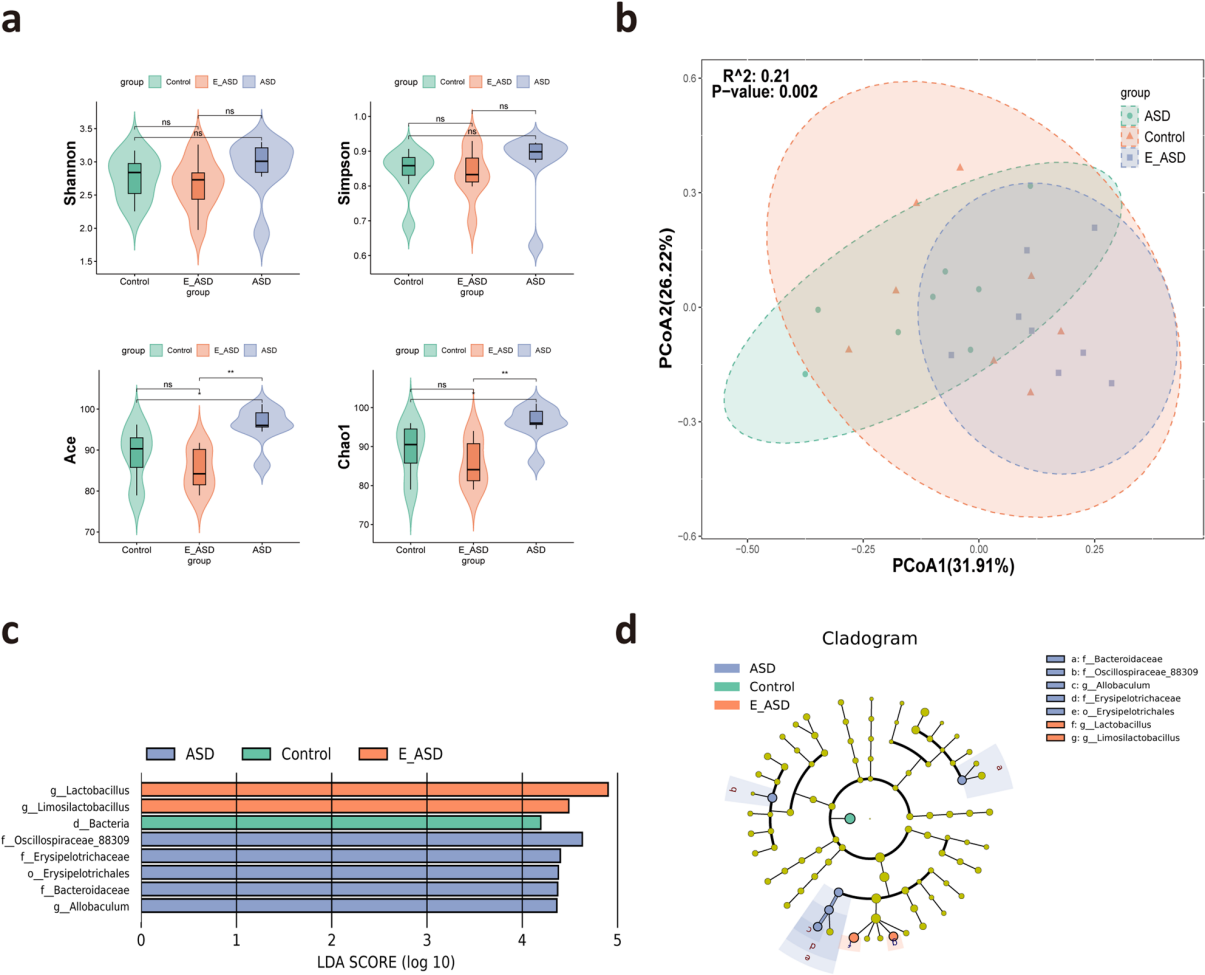
Comparative analysis of the gut microbiome across the control, ASD, and E_ASD groups. **a**. Alpha diversity indices, including the Shannon, Simpson, ACE, and Chao1 indices, for the control, ASD, and E_ASD groups; **b**. PCoA plot based on Bray-Curtis distances, showing the separation of the gut microbiome profiles among the control, ASD, and E_ASD groups, with R^2^ = 0.21 and *p* value = 0.002 indicating the significance of the group separation; **c**. LEfSe of the gut microbiota, displaying taxa with significant differences in relative abundance between groups, with LDA scores highlighting the importance of each taxon in distinguishing between the groups; **d**. Cladogram illustrating the phylogenetic relationships between differentially abundant taxa identified by LEfSe analysis, with branch tips colored according to group abundance. N = 8 (male= 4, female = 4)

To investigate whether exercise affects ASD behavior through changes in the gut microbiota, SCFAs, and neurotransmitters, we measured these three indicators. In the control group (Fig. 2a), the gut microbial community was characterized by a dominant taxa profile enriched with typical beneficial genera such as *Ligilactobacillus* and *Lactobacillus*, reflecting a stable microbial structure associated with healthy gut physiology. In contrast, the ASD group (Fig. 2b) exhibited a distinct microbial signature, with *Bacteroides* and *Prevotella* constituting the major dominant taxa, representing a characteristic ASD-associated microbial configuration. The microbiota composition of the E_ASD group (Fig. 2c) showed a shift away from the ASD-like pattern, indicating partial improvement in microbial imbalance. The α diversity results revealed no significant differences between the Shannon and Simpson indices. However, consistent patterns were observed for the ACE and Chao1 indices, indicating that the species diversity in the ASD group was significantly greater than that in both the E_ASD and control groups (Fig. 3a). Notably, no significant changes were observed between the E_ASD and control groups. For β-diversity analysis, PCoA based on Bray-Curtis distances revealed distinct clustering patterns among the three groups, further supported by PERMANOVA, which confirmed significant differences in the gut microbiota composition (Fig. 3b). Lefse analysis provided insights into specific taxonomic enrichments within each group (Fig. 3c, d). Notably, the E_ASD group was enriched with *Lactobacillus* and *Limosilactobacillus* at the genus level. Interestingly, the ASD group presented an overabundance of *Allobaculum*.

**Fig. 4:**
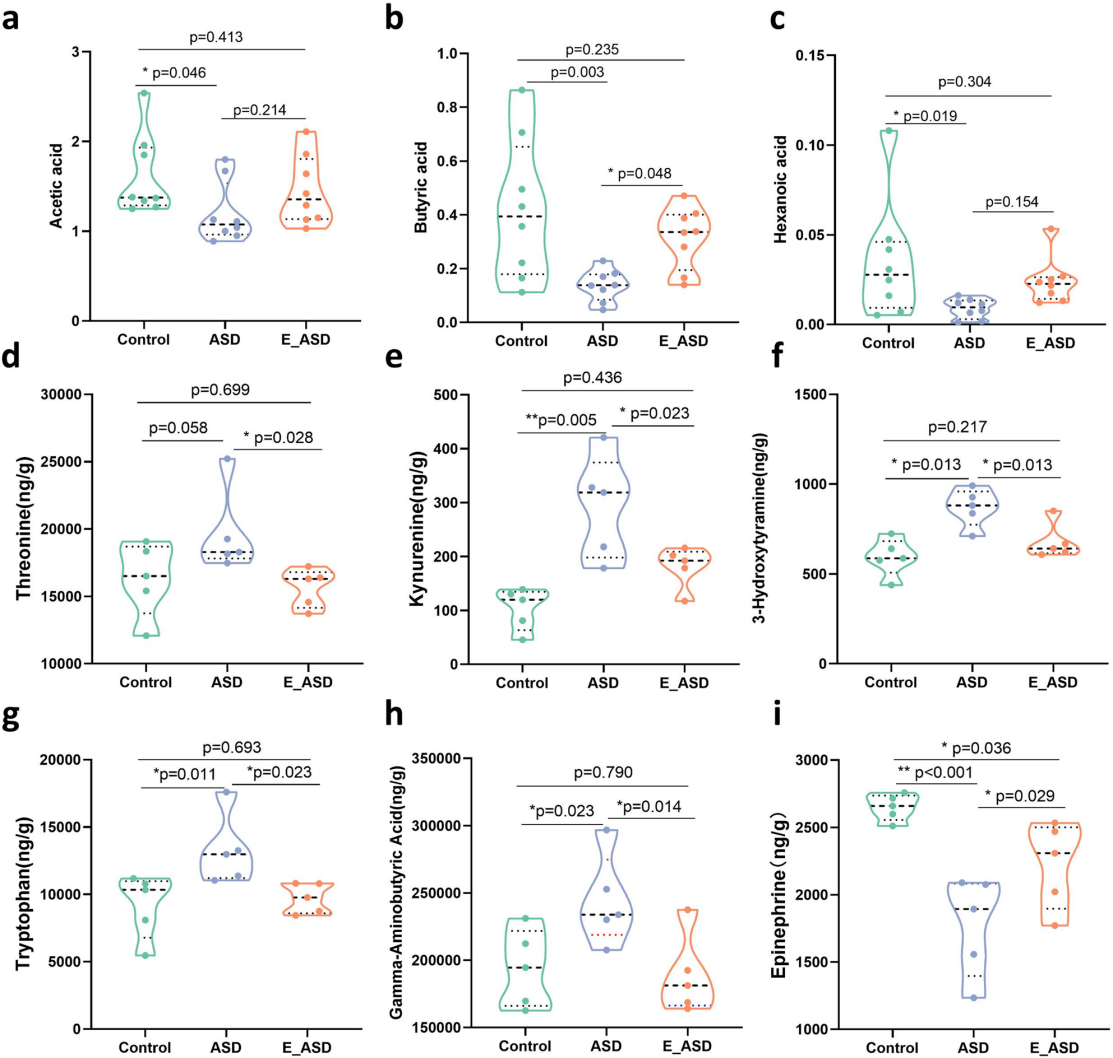
Metabolic profile comparisons among the control, ASD, and E_ASD groups. **a**. Concentrations of acetic acid; **b**. Concentrations of butyric acid; **c**. Concentrations of hexanoic acid; **d**. Concentrations of threonine; **e**. Concentrations of kynurenine; **f**. Concentrations of 3-hydroxykynurenine; **g**. Concentrations of tryptophan; **h**. Concentrations of gamma-aminobutyric acid; **i**. Concentrations of epinephrine. *p<0.05, **p<0.01 indicate significant differences between groups. N_a-c_ = 8 (male = 4, female = 4), N_d-i_ = 5

Furthermore, we found that butyrate levels were significantly lower in the ASD group compared with both the E_ASD and control groups, while no significant difference was observed between the E_ASD and control groups (Fig. 4b). The levels of acetic acid and hexanoic acid were significantly lower in the ASD group than in the control group, while the levels in the E_ASD group tended to increase, although the difference was not statistically significant (Fig. 4a, c).

Importantly, neurotransmitter analysis of the prefrontal cortex (PFC) of the E_ASD rats revealed significant reductions in threonine (Fig. 4d), kynurenine (Fig. 4e), 3-hydroxytyramine (Fig. 4f), tryptophan (Fig. 4g), and gamma-aminobutyric acid (Fig. 4h) levels compared with those in the ASD group, with no significant differences compared with those in the control group. Epinephrine levels were significantly greater in the E_ASD group than in the ASD group (Fig. 4i), approaching those of the control group but still showing a notable difference.

### FMT from E_ASD group rats alleviates autism-like behavior

**Fig. 5:**
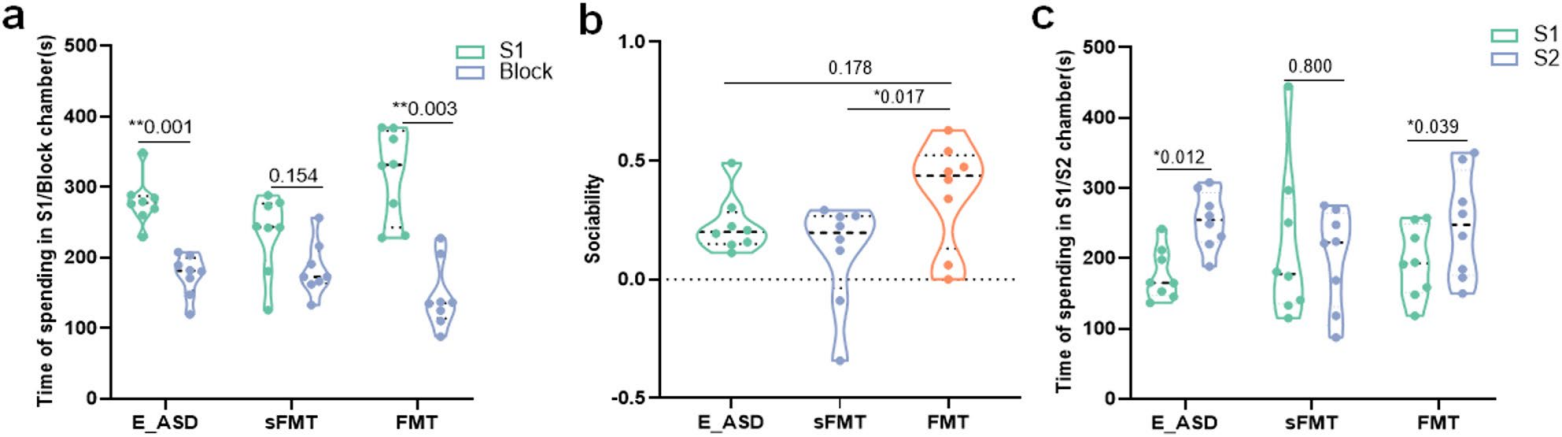
Behavioral assessments across three groups: the E_ASD, FMT and sFMT groups. **a**. Time spent in the stranger-1 (S1) chamber versus the empty chamber (Block) during the sociability phase. **b**. Sociability index calculated as (S1 − Block) / (S1 + Block). **c**. Time spent in the stranger-1 (S1) and stranger-2 (S2) chambers during the social novelty preference phase. *p < 0.05, **p < 0.01 indicate significant differences between groups. *N* =8 (male = 4, female = 4)

Exercise-induced gut microbiota involvement in behavioral improvement was assessed by performing fecal microbiota transplantation from the E_ASD group to ASD rats, with sFMT used as the control (Fig. S1b). After four weeks of intervention, the FMT group exhibited clear behavioral improvements in the three-chamber social test (Fig. 5a–c). During the sociability phase (S1 vs. Block), rats in the FMT group spent significantly more time in the stranger-1 (S1) chamber than in the empty chamber (Fig. 5a), demonstrating a clear restoration of social interest. In contrast, the sFMT group did not show a significant difference between the two chambers. Consistently, the sociability index of the FMT group was significantly higher than that of the sFMT group (Fig. 5b), further supporting the beneficial effect of FMT on sociability. During the social novelty preference phase (S1 vs. S2), the FMT group spent significantly more time in the stranger-2 (S2) chamber than in the S1 chamber, demonstrating a distinct preference for social novelty, whereas the sFMT group showed no such preference (Fig. 5c).

### FMT alters the gut microbiota, SCFAs, and neurotransmitters in ASD rats

**Fig. 6:**
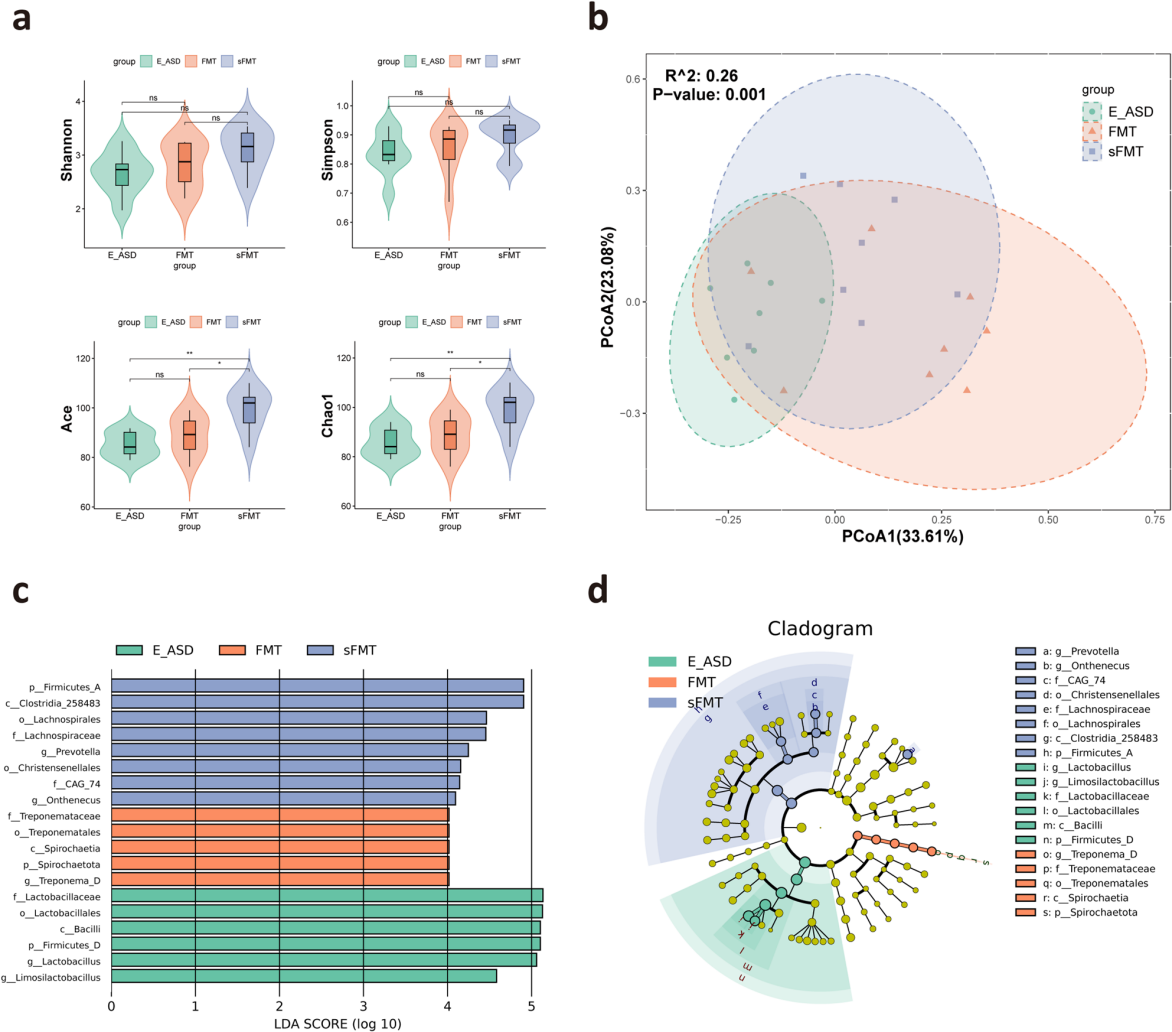
Comparative analysis of the gut microbiome across the E_ASD, FMT, and sFMT groups. **a**. Alpha diversity indices, including the Shannon, Simpson, ACE, and Chao1 indices, for the E_ASD, FMT, and sFMT groups; **b**. PCoA plot based on Bray-Curtis distances, showing the separation of the gut microbiome profiles among the E_ASD, FMT, and sFMT groups, with R^2^ = 0.26 and *p* value = 0.001 indicating the significance of the group separation; **c**. LEfSe of the gut microbiota, displaying taxa with significant differences in relative abundance between groups, with LDA scores highlighting the importance of each taxon in distinguishing between the groups; **d**. Cladogram illustrating the phylogenetic relationships between differentially abundant taxa identified by LEfSe analysis, with branch tips colored according to group abundance. N = 8 (male = 4, female = 4)

After four weeks of FMT, significant changes were observed in the gut microbiota, SCFA levels, and neurotransmitter profiles of the ASD rats. Notably, the sFMT group presented minimal differences compared with the previous ASD group (Fig. 2e), whereas the FMT group presented increases in the *Turicibacter* and *Clostridium* genera (Fig. 2d). Moreover, significant alterations in the α diversity of the gut microbiota were detected across all groups (Fig. 6a). Notably, compared with both the E_ASD and FMT groups, the sFMT group presented significant differences in gut microbial diversity, as observed in both the Chao1 and ACE indices. However, no significant differences in α diversity were found between the E_ASD and FMT groups. Additionally, β diversity analysis revealed differences in the gut microbiota composition among the three groups (Fig. 6b). Importantly, Bray–Curtis PCoA revealed a pronounced separation between the FMT and ASD groups (Fig. S4), indicating that exercise-derived microbiota effectively reshaped the recipient microbial community. In contrast, no separation was observed between the sFMT and ASD groups (Fig. S5), confirming that saline gavage alone did not alter the native microbial structure. Lefse analysis revealed distinct microbial taxa between these groups (Fig. 6c, d). The E_ASD group exhibited continued enrichment of *Lactobacillus* and *Limosilactobacillus*, whereas the sFMT group presented increased abundances of the *Prevotella* and *Onthenecus* genera, and the FMT group was enriched in *Treponema*.

**Fig. 7:**
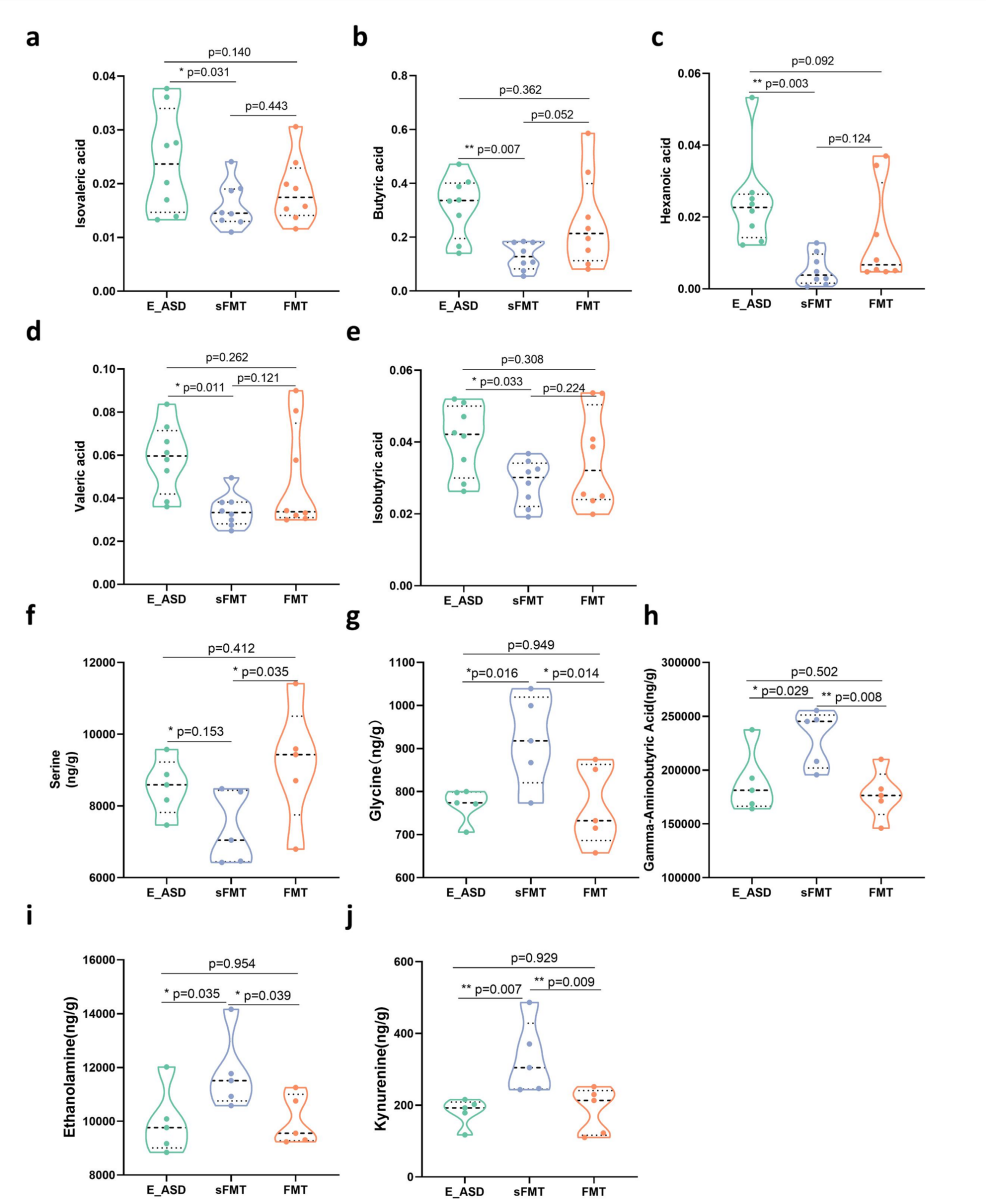
Metabolic profile comparisons among the E_ASD, FMT, and sFMT groups. **a**. Concentrations of isovaleric acid; **b**. Concentrations of butyric acid; **c**. Concentrations of hexanoic acid; **d**. Concentrations of valeric acid; **e**. Concentrations of isobutyric acid; **f**. Concentrations of serine; **g**. Concentrations of glycine; **h**. Concentrations of gamma-aminobutyric acid; **i**. Concentrations of ethanolamine; **j**. Concentrations of kynurenine. *p < 0.05, **p < 0.01 indicate significant differences between groups, with specific comparisons detailed in the figure. N_a-e_ = 8 (male = 4, female = 4), N_f-j_ = 5

In terms of SCFAs, we observed significant reductions in the levels of isovaleric acid (Fig. 7a), butyric acid (Fig. 7b), hexanoic acid (Fig. 7c), valeric acid (Fig. 7d), and isobutyric acid (Fig. 7e) in the sFMT group compared with those in the E_ASD group. However, no significant differences were found between the FMT and E_ASD groups. Although an increasing trend in SCFA levels was observed in the FMT group compared with the sFMT group, this difference was not statistically significant.

Interestingly, neurotransmitter analysis revealed a significant increase in serine (Fig. 7f) in the FMT group compared with the sFMT group. In addition, glycine, γ-aminobutyric acid, ethanolamine, and kynurenine levels in the FMT group closely resembled those in the E_ASD group, with no significant differences between the two. Notably, both groups exhibited significantly lower levels than the sFMT group, indicating that FMT restored these neurotransmitters abnormalities toward the profile observed after exercise intervention (Fig. 7g-j).

### Correlations between the differential SCFA, neurotransmitter and bacterial taxa

**Fig. 8:**
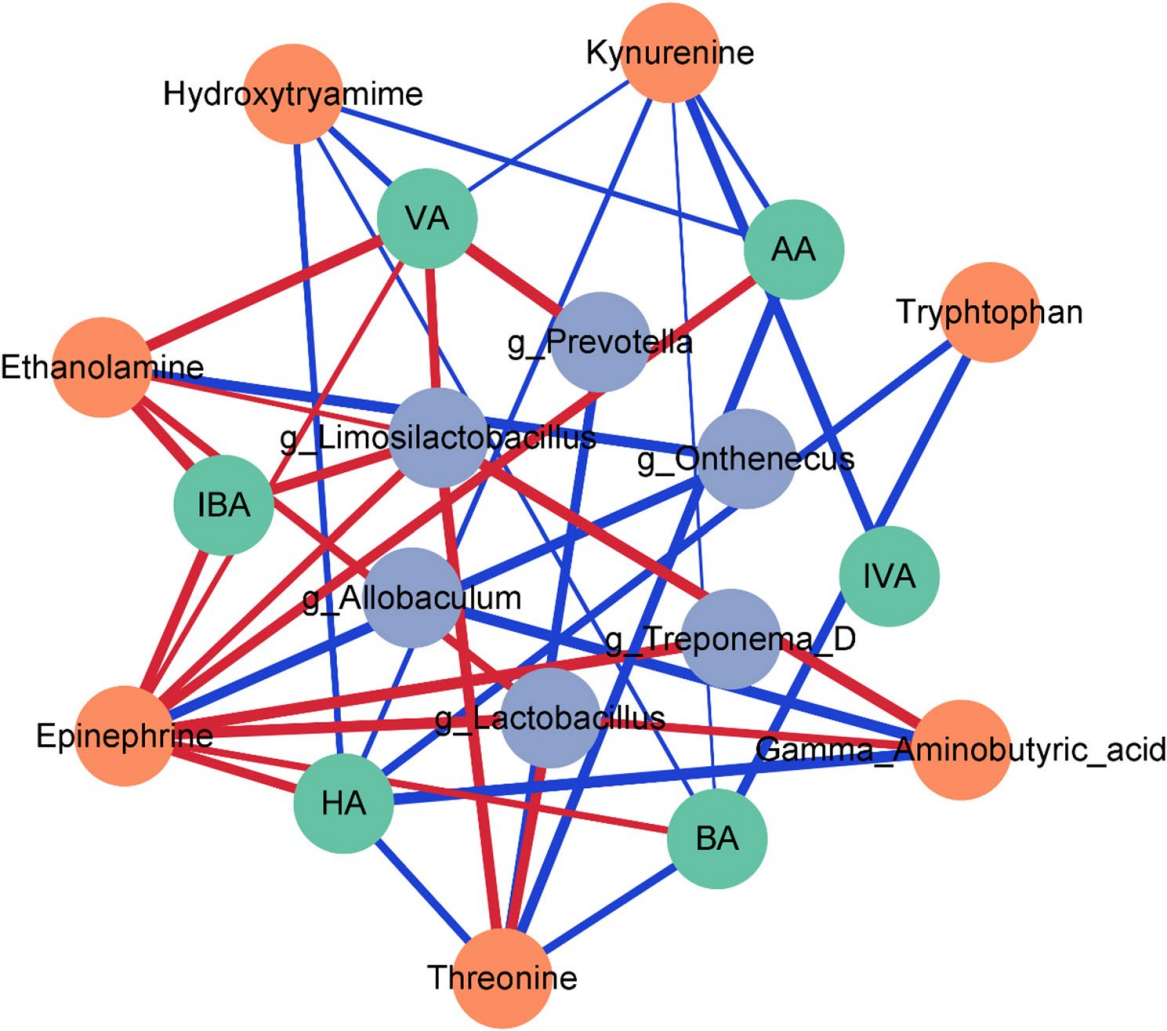
Network analysis of microbiota‒metabolite interactions in the E_ASD group. This network diagram illustrates the interactions between the gut microbiota and metabolites. The blue nodes represent bacterial genera, the green nodes represent neurotransmitters and other related compounds, and the orange nodes represent SCFAs and other metabolites. The connections between nodes indicate correlations or interactions, with the thickness of the lines reflecting the strength of the relationships. Red edges represent positive correlations, whereas blue edges indicate negative correlations. AA, Acetic acid; IBA, Isobutyric acid; BA, Butyric acid; IVA, Isovaleric acid; VA, Valeric acid; HA, Hexanoic acid

A correlation network was constructed to examine the interactions among differential bacterial taxa, SCFAs, and neurotransmitters (Fig. 8). The adjusted network contained 19 nodes and 38 significant edges, with nodes representing specific taxa or metabolites and edges indicating statistically significant correlations. Among the bacterial taxa, *Prevotella*, *Lactobacillus*, and *Limosilactobacillus* exhibited high connectivity, each forming multiple associations with SCFAs and neurotransmitters, suggesting their prominent roles within the microbial–metabolic interaction landscape.

Among the metabolites, epinephrine emerged as the most highly connected node, displaying strong positive correlations with several bacterial taxa, whereas kynurenine showed predominantly negative correlations with multiple neurotransmitters, indicating its distinct regulatory pattern within the metabolic network. These interaction patterns highlight key microbial and metabolic nodes that may contribute to the regulation of the gut-brain metabolic axis.

## Discussion

Over the past years, research has increasingly illuminated the impact of exercise on the gut microbiota [35–40], with an increasing number of studies confirming the link between gut microbes and autism. This study investigated the effects of six weeks of voluntary wheel running on autism-like behaviors and its association with changes in the gut microbiota. The results demonstrated that this six-week exercise intervention not only ameliorated autism-like behaviors but also significantly modulated the gut microbiota, SCFAs, and neurotransmitters in the PFC. Moreover, transplantation of gut microbiota from the E_ASD group markedly improved behavioral performance in ASD rats, accompanied by alterations in SCFAs and neurotransmitter levels. These findings suggest that exercise intervention improves autism-like behaviors by modulating the gut microbiota, which in turn influences SCFAs and neurotransmitters.

From a behavioral perspective, early-stage exercise primarily improved social interest and preference for social novelty in VPA rats, while having no significant effect on anxiety-like behavior in the open field. This pattern of “social behavior improvement taking precedence” aligns with previous research, indicating that exercise more readily modulates prefrontal-limbic circuits associated with social motivation and reward processing, while exerting relatively limited influence on anxiety- or emotion-related amygdala pathways [41–44]. Previous research suggests exercise enhances synaptic plasticity in the PFC and boosts signaling of reward-related neurotransmitters [45]. Notably, gut microbiota transplantation in the exercise group partially replicated the improvement in social behavior, whereas no similar changes were observed in the sFMT group. These findings indicate that exercise-induced microbial changes can transfer social behavioral benefits, consistent with the gut–brain axis as a key regulator of social function.

Our findings indicated that six weeks of voluntary wheel running led to alterations in the gut bacterial profiles, notably increasing the abundance of the genera *Limosilactobacillus* and *Lactobacillus*, which are recognized as probiotics involved in carbohydrate fermentation into lactic acid and butyric acid. Recent studies have shown that *Limosilactobacillus reuteri* significantly improves social function in children with ASD, with positive effects observed across various measurement indicators. However, it does not significantly affect the overall severity of autism or other behavioral domains [46]. In addition, *Lactobacillus plantarum PS128* has also demonstrated significant improvements in children with ASD, particularly in social communication, with fewer side effects. Research indicates that younger children benefit more prominently, highlighting the specific effects of these strains in alleviating ASD symptoms [47], particularly in the improvement of social behaviors. These findings support the notion that exercise may enhance digestion and overall gut health by promoting the growth of beneficial bacteria [48–52], suggesting that exercise plays a role in fostering a healthier gut microbiome.

Our study also revealed the positive impact of exercise on the production of SCFAs by the microbiota. Previous studies have demonstrated that exercise can modulate gut microbiota and their metabolites, particularly short-chain fatty acids, and exert effects on central nervous system function through multiple gut-brain pathways-including immune signaling, the vagus nerve, and the HPA axis-acting in concert. This mechanistic framework provides a crucial foundation for understanding how exercise improves brain function [53]. Specifically, exercise was found to increase the levels of butyric acid, acetic acid, and hexanoic acid, which are associated with various health benefits, including enhanced immune function, improved colonic epithelial cell integrity, and improved brain function [54]. These findings align with those of previous studies, such as the work of Matsumoto et al. [55], who reported an increase in butyric acid levels in the cecal contents of rats following 5 weeks of voluntary wheel running. Similarly, Erlandson et al. [56] reported that sedentary elderly individuals presented increased butyric acid levels in the gut following exercise intervention. Additionally, Barton et al. [54] reported that athletes had higher levels of SCFAs than did sedentary individuals. Notably, our previous research revealed that, compared with control rats, rats with VPA-induced ASD presented lower SCFA levels in their feces [33]. However, in the present study, we found that six weeks of voluntary wheel running significantly increased SCFA levels in the feces of autistic rats, restoring them to within the normal range. SCFAs play a critical role in the growth and development of both the intestinal and central systems, serving as essential energy substrates [57]. Notably, accumulating evidence also supports the view that gut microbes influence central neurochemistry [14].

Moreover, previous studies have suggested that a neuronal excitation/inhibition imbalance caused by dysfunctional neurotransmitters is an important etiology of autism [57–59] and that gut microbes are capable of producing most neurotransmitters found in the human brain [14]. A cohort study revealed that multiple neurotransmitter biosynthesis-related pathways in the gut microbiome were depleted in children with ASD compared with TD children [60]. Therefore, in this study, we further measured 55 transmitters in the PFC through neurotransmitter-targeting technology via LC‒MS. We found that, in the early stage of improvement during adolescence, several neurotransmitters in the PFC, such as threonine, kynurenine, tryptophan, 5-hydroxyindoleacetic acid, and betaine aldehyde chloride [33], were present at lower levels in the ASD group than in the control group. However, at adulthood, after 10 weeks, the levels of these neurotransmitters were greater than those in the control group. Notably, the trend of neurotransmitter changes in the exercised group was consistent with that in the control group, indicating a restorative effect. These findings support existing research suggesting that brain development may be associated with the characteristics of ASD. In summary, this study demonstrated that exercise can ameliorate the behavioral and synaptic abnormalities in offspring induced by VPA exposure, providing potential insights for interventions in ASD.

At the molecular level, exercise-induced changes in SCFAs and neurotransmitters may jointly influence ASD-like behaviors through multiple intersecting pathways. As a prototypical anti-inflammatory metabolite, butyrate not only enhances gene expression of neuroplasticity-related genes in the PFC and hippocampus but also improves intestinal barrier function and reduces peripheral inflammation, thereby diminishing inflammatory signaling interference with the central nervous system [61–63]. Acetic acid and propionic acid, meanwhile, transmit gut signals to the brainstem and limbic system via the vagus nerve, modulating neural circuits associated with social motivation [64]– [65]. At the same time, exercise-induced reshaping of tryptophan metabolism may reduce the production of neurotoxic metabolites such as 3-hydroxykynurenine and enhance serine-related signaling, a process typically associated with reduced oxidative stress and alleviation of autism-like symptoms [66]– [67]. Collectively, exercise synergistically optimizes gut-brain axis function across multiple levels by modulating microbial metabolites and brain neurotransmitters, thereby producing comprehensive improvements in ASD-like behaviors.

More importantly, to investigate the causal relationship between behavioral changes and the effects of exercise on the microbiota-gut-brain axis, we transplanted the fecal microbiota from the E_ASD group rats into the ASD group rats via intragastric administration. The results indicated that after four weeks of FMT, the FMT group rats presented significant behavioral improvements, along with alterations in the gut microbiota, SCFAs, and central neurotransmitters, which began to resemble the profiles observed in the E_ASD group. However, no significant changes were observed in the sFMT group. At present, the commonly used methods are beneficial bacterial supplementation, diet structure adjustment, antibiotic treatment and fecal bacterial transplantation. Although some small-scale studies have suggested that probiotic supplementation may help alleviate certain complications of ASD, such as constipation, or reduce its core symptoms, the benefits observed are generally modest and short-lived. This may be because supplementation with a certain probiotic alone has too little effect on the intestinal microecology. For example, a systematic review by Kristensen et al. [68] revealed that supplementation with probiotics did not seem to affect the composition of the fecal microbiota in healthy people. Microecotherapy is considered a very promising protocol for targeting related neurodevelopmental diseases such as ASD [69, 70]. The results of the population experiment by Lin et al. [71] also showed that transplanting the gut microbiota of normally developing children into ASD patients could significantly improve their gut microbiota properties. In 2017, Kang et al. [72] reported the transplantation of the gut microbiota in children with ASD complicated with gastrointestinal problems and reported that the intestinal problems of patients were not only reduced but also that the core symptoms of ASD improved. Moreover, the results of the follow-up two years later showed that this improvement effect still persisted [10]. Recently, Chen et al. [73] transplanted fecal bacteria from healthy people into ASD mice after in vitro culture (gavage once every other day, a total of 7 times) and reported that the core symptoms of ASD model mice could be significantly improved. These findings suggest that supplementing with the body’s native gut microbiota, as a source of regulation, may offer more durable and effective results compared to supplementation with exogenous microbes, such as most current probiotics, which are not isolated from the gut. The adjustment of the gut microbiota by exercise is driven by exercise and does not produce exogenous microorganisms; thus, the gut microbiota can be well colonized and function after transplantation into the same type of body. Therefore, in the present study, we transplanted the whole gut microbiota of the exercise group. Notably, after 4 weeks of FMT, incomplete alignment was observed in the FMT group and the E_ASD group after transplantation. This discrepancy can be attributed to two factors: the interactions among the gut microbiota itself and the interactions among neurotransmitters. However, further studies are needed to clarify the specific mechanisms involved.

Notably, while both exercise intervention and FMT improve ASD-like behaviors, the alterations they induce in gut microbiota composition, SCFA levels, and neurotransmitter profiles are not entirely consistent. This suggests their effects may not stem from a simple “species replication” of donor microbes, but rather represent a function-centered “selective expression” process [74]. Despite differing taxonomic compositions, both interventions were accompanied by restorative regulation of key SCFAs like butyrate and multiple neurotransmitter pathways. This function-priority expression pattern aligns more closely with the homeostasis principles of the gut ecosystem and explains why FMT can only partially replicate the neurochemical effects triggered by exercise intervention. Overall, this phenomenon further underscores the high complexity and multi-pathway synergistic mechanisms of the gut-brain axis across various diseases [75]– [76].

Although this study provides preliminary evidence suggesting that exercise can regulate various central neurotransmitters in ASD rats through the gut microbiota, several limitations should be considered. One limitation of this study is the relatively small sample size, which may affect the generalizability of the results. Additionally, although 16S rRNA gene sequencing provides valuable information about the composition of the gut microbiota, it primarily identifies microorganisms at the genus level and may not fully capture the specific microbial species involved in modulating ASD-like behaviors. Furthermore, this study focused on short-term interventions (6 weeks of exercise and 4 weeks of FMT), and the long-term effects of exercise and microbiota modulation remain unclear. The lack of follow-up behavioral assessments after the interventions also limits our understanding of the sustained impact of exercise on ASD-like behaviors. Finally, while the results support the role of gut microbiota and short-chain fatty acids in behavior modulation, the specific molecular mechanisms underlying these effects require further investigation.

## Conclusions

In conclusion, our study demonstrated that voluntary wheel running significantly improved the behavioral performance of rats with ASD. It not only increases the abundance of *Limosilactobacillus* and *Lactobacillus* but also modulates the levels of SCFAs and neurotransmitters, bringing them closer to the levels observed in normal rats. When the gut microbiota from the exercise group was transplanted into ASD rats, similar changes were observed as those in the exercise group. These findings suggest that physical exercise and FMT may influence SCFAs and neurotransmitters by modulating the gut microbiota, providing a potential mechanism for the improvement of ASD-like behaviors.

## Supplementary Information

Supplementary Material 1.

## Data Availability

All data generated and/or analyzed in this study have been deposited in the Genome Sequence Archive (GSA). The raw sequencing data can be retrieved using the GSA accession number CRA029953, and the project-level information corresponds to the BioProject accession PRJCA046171.

## References

[1] American Psychiatric Association. Diagnostic and Statistical Manual of Mental Disorders (DSM-5). 5th ed. Washington, DC: American Psychiatric Association; 2013.

[2] Maenner MJ, Warren Z, Williams AR, et al. Prevalence and characteristics of autism spectrum disorder among children aged 8 Years - autism and developmental disabilities monitoring Network, 11 Sites, united States, 2020[J]. MMWR Surveill Summ. 2023;72(2):1–14. 36952288 10.15585/mmwr.ss7202a1 PMC10042614

[3] Cryan JF, O’riordan KJ, Cowan CSM, et al. The Microbiota-Gut-Brain Axis[J]. Physiol Rev. 2019;99(4):1877–2013. 31460832 10.1152/physrev.00018.2018

[4] Pulikkan J, Mazumder A, Grace T. Role of the gut Microbiome in autism spectrum Disorders[J]. Adv Exp Med Biol. 2019;1118:253–69. 30747427 10.1007/978-3-030-05542-4_13

[5] Xu M, Xu X, Li J, et al. Association between gut microbiota and autism spectrum disorder: A systematic review and Meta-Analysis[J]. Front Psychiatry. 2019;10:473. 31404299 10.3389/fpsyt.2019.00473 PMC6673757

[6] Hughes HK, Rose D, Ashwood P. The gut microbiota and dysbiosis in autism spectrum Disorders[J]. Curr Neurol Neurosci Rep. 2018;18(11):81. 30251184 10.1007/s11910-018-0887-6 PMC6855251

[7] Navya Bezawada, Tze Hui Phang, Georgina L. Hold, Richard Hansen; Autism Spectrum Disorder and the Gut Microbiota in Children: A Systematic Review. Ann Nutr Metab. 2020;76(1):16–29. 10.1159/000505363. 10.1159/000505363 31982866

[8] Ding H, Yi X, Zhang X, et al. Imbalance in the gut microbiota of children with autism spectrum Disorders[J]. Front Cell Infect Microbiol. 2021;11:572752. 34790583 10.3389/fcimb.2021.572752 PMC8591234

[9] Sharon G, Cruz NJ, Kang DW, et al. Human gut microbiota from autism spectrum disorder promote behavioral symptoms in Mice[J]. Cell. 2019;177(6):1600–e161817. 31150625 10.1016/j.cell.2019.05.004 PMC6993574

[10] Kang DW, Adams JB, Coleman DM, et al. Long-term benefit of microbiota transfer therapy on autism symptoms and gut microbiota[J]. Sci Rep. 2019;9(1):5821. 30967657 10.1038/s41598-019-42183-0 PMC6456593

[11] Li Q, Zhou JM. The microbiota-gut-brain axis and its potential therapeutic role in autism spectrum disorder[J]. Neuroscience. 2016;324:131–9. 26964681 10.1016/j.neuroscience.2016.03.013

[12] Fowlie G, Cohen N, Ming X. The Perturbance of Microbiome and Gut-Brain Axis in Autism Spectrum Disorders. Int J Mol Sci. 2018;19(8):2251. 10.3390/ijms19082251. 10.3390/ijms19082251 PMC6121241 30071612

[13] Abdel-Haq R, Schlachetzki JCM, Glass CK, et al. Microbiome-microglia connections via the gut-brain axis[J]. J Exp Med. 2019;216(1):41–59. 30385457 10.1084/jem.20180794 PMC6314531

[14] Dinan TG, Cryan JF. The Microbiome-Gut-Brain axis in health and Disease[J]. Gastroenterol Clin North Am. 2017;46(1):77–89. 28164854 10.1016/j.gtc.2016.09.007

[15] Yang Y, Tian J, Yang B. Targeting gut microbiome: A novel and potential therapy for autism[J]. Life Sci. 2018;194:111–9. 29277311 10.1016/j.lfs.2017.12.027

[16] Lambert JE, Myslicki JP, Bomhof MR, et al. Exercise training modifies gut microbiota in normal and diabetic mice[J]. Appl Physiol Nutr Metab. 2015;40(7):749–52. 25962839 10.1139/apnm-2014-0452

[17] Abraham D, Feher J, Scuderi GL, et al. Exercise and probiotics attenuate the development of alzheimer’s disease in Transgenic mice: role of microbiome[J]. Exp Gerontol. 2019;115:122–31. 30529024 10.1016/j.exger.2018.12.005

[18] Allen JM, Mailing LJ, Cohrs J, et al. Exercise training-induced modification of the gut microbiota persists after microbiota colonization and attenuates the response to chemically-induced colitis in gnotobiotic mice[J]. Gut Microbes. 2018;9(2):115–30. 28862530 10.1080/19490976.2017.1372077 PMC5989796

[19] Campbell SC, Wisniewski PJ, Noji M, et al. The effect of diet and exercise on intestinal integrity and microbial diversity in Mice[J]. PLoS ONE. 2016;11(3):e0150502. 26954359 10.1371/journal.pone.0150502 PMC4783017

[20] Ferreira JP, Ghiarone T, Cabral Júnior CR, Furtado GE, Moreira Carvalho H, Machado-Rodrigues AM, Andrade Toscano CV. Effects of Physical Exercise on the Stereotyped Behavior of Children with Autism Spectrum Disorders. Medicina. 2019;55(10):685. 10.3390/medicina55100685. 10.3390/medicina55100685 PMC6843401 31615098

[21] Wang J-G, Cai K-L, Liu Z-M, Herold F, Zou L, Zhu L-N, Xiong X, Chen A-G. Effects of Mini-Basketball Training Program on Executive Functions and Core Symptoms among Preschool Children with Autism Spectrum Disorders. Brain Sci. 2020;10(5):263. 10.3390/brainsci10050263. 10.3390/brainsci10050263 PMC7287705 32365853

[22] Cai K, Yu Q, Herold F, et al. Mini-Basketball training program improves social communication and white matter integrity in children with Autism[J]. Brain Sci. 2020;10(11):1–14. 10.3390/brainsci10110803 PMC7693206 33142694

[23] Liang X, Li R, Wong SHS, et al. The effects of exercise interventions on executive functions in children and adolescents with autism spectrum disorder: A systematic review and Meta-analysis[J]. Sports Med. 2022;52(1):75–88. 34468951 10.1007/s40279-021-01545-3

[24] Healy S, Nacario A, Braithwaite RE, et al. The effect of physical activity interventions on youth with autism spectrum disorder: A meta-analysis[J]. Autism Res. 2018;11(6):818–33. 29693781 10.1002/aur.1955

[25] Tan BW, Pooley JA, Speelman CP. A Meta-Analytic review of the efficacy of physical exercise interventions on cognition in individuals with autism spectrum disorder and ADHD[J]. J Autism Dev Disord. 2016;46(9):3126–43. 27412579 10.1007/s10803-016-2854-x

[26] Bremer E, Crozier M, Lloyd M. A systematic review of the behavioural outcomes following exercise interventions for children and youth with autism spectrum disorder[J]. Autism. 2016;20(8):899–915. 26823546 10.1177/1362361315616002

[27] Wang S, Chen D, Yang Y, et al. Effectiveness of physical activity interventions for core symptoms of autism spectrum disorder: A systematic review and meta-analysis[J]. Autism Res. 2023;16(9):1811–24. 37539450 10.1002/aur.3004

[28] Pietrelli A, Matkovic L, Vacotto M, et al. Aerobic exercise upregulates the BDNF-Serotonin systems and improves the cognitive function in rats[J]. Neurobiol Learn Mem. 2018;155:528–42. 29800645 10.1016/j.nlm.2018.05.007

[29] Bequet F, Gomez-Merino D, Berthelot M, et al. Exercise-induced changes in brain glucose and serotonin revealed by Microdialysis in rat hippocampus: effect of glucose supplementation[J]. Acta Physiol Scand. 2001;173(2):223–30. 11683680 10.1046/j.1365-201X.2001.00859.x

[30] Alizadeh Pahlavani H. Possible role of exercise therapy on depression: effector neurotransmitters as key players[J]. Behav Brain Res. 2023;459:114791. 38048912 10.1016/j.bbr.2023.114791

[31] Ercan Z, Bulmus O, Kacar E, et al. Treadmill exercise improves behavioral and Neurobiological alterations in Restraint-Stressed Rats[J]. J Mol Neurosci. 2023;73(9–10):831–42. 37794307 10.1007/s12031-023-02159-2

[32] Schneider T, Przewłocki R. Behavioral alterations in rats prenatally exposed to valproic acid: animal model of autism[J]. Neuropsychopharmacology. 2005;30(1):80–9. 15238991 10.1038/sj.npp.1300518

[33] Zhong JG, Lan WT, Feng YQ, et al. Associations between dysbiosis gut microbiota and changes of neurotransmitters and short-chain fatty acids in valproic acid model rats[J]. Front Physiol. 2023;14:1077821. 37035670 10.3389/fphys.2023.1077821 PMC10073564

[34] Sampson TR, Debelius JW, Thron T, et al. Gut microbiota regulate motor deficits and neuroinflammation in a model of parkinson’s Disease[J]. Cell. 2016;167(6):1469–e148012. 27912057 10.1016/j.cell.2016.11.018 PMC5718049

[35] Zhong F, Wen X, Yang M, et al. Effect of an 8-week exercise training on gut microbiota in physically inactive older Women[J]. Int J Sports Med. 2021;42(7):610–23. 33321523 10.1055/a-1301-7011

[36] Yang J, Wu JE, Li Y, Zhang YE, Cho WC, Ju X, Zheng Y. Gut bacteria formation and influencing factors. FEMS Microbiol Ecol. 2021;97(4):fiab043. 10.1093/femsec/fiab043. 10.1093/femsec/fiab043 33705527

[37] Wang G, Zhou HH, Luo L, et al. Voluntary wheel running is capable of improving cognitive function only in the young but not the middle-aged male APPSwe/PS1De9 mice[J]. Neurochem Int. 2021;145:105010. 33684544 10.1016/j.neuint.2021.105010

[38] Verheggen R, Konstanti P, Smidt H, et al. Eight-week exercise training in humans with obesity: marked improvements in insulin sensitivity and modest changes in gut microbiome[J]. Obes (Silver Spring). 2021;29(10):1615–24. 10.1002/oby.23252 PMC9291576 34467673

[39] Koutouratsas T, Philippou A, Kolios G, et al. Role of exercise in preventing and restoring gut dysbiosis in patients with inflammatory bowel diseases: A review[J]. World J Gastroenterol. 2021;27(30):5037–46. 34497433 10.3748/wjg.v27.i30.5037 PMC8384738

[40] Campbell SC, Wisniewski PJ. 2nd. Exercise is a novel promoter of intestinal health and microbial Diversity[J]. Exerc Sport Sci Rev. 2017;45(1):41–7. 27782912 10.1249/JES.0000000000000096

[41] Ochi R, Fujita N, Takaishi K, Oshima T, Nguyen ST, Nishijo H, et al. Voluntary exercise reverses social behavior deficits and the increases in the densities of cholecystokinin-positive neurons in specific corticolimbic regions of diabetic OLETF rats. Behav Brain Res. 2022;428:113886. 10.1016/j.bbr.2022.113886. 35398486 10.1016/j.bbr.2022.113886

[42] Goodpaster CM, Christensen CR, Alturki M-B, DeNardo LA. Prefrontal cortex development and its implications in mental illness. Neuropsychopharmacology. 2026;51:114–28. 10.1038/s41386-025-02154-8. 40603750 10.1038/s41386-025-02154-8 PMC12618524

[43] Taguchi S, Choudhury ME, Mikami K, Utsunomiya R, Yano H, Tanaka J. Treadmill exercise as a preventive measure against Age-Related anxiety and social behavioral disorders in rats: when is it worth starting? Ann Rehabil Med. 2022;46:320–8. 10.5535/arm.22105. 36588447 10.5535/arm.22105 PMC9810656

[44] Siuda J, Patalong-Ogiewa M, Żmuda W, Targosz-Gajniak M, Niewiadomska E, Matuszek I, et al. Cognitive impairment and BDNF serum levels. Neurol Neurochir Pol. 2017;51:24–32. 10.1016/j.pjnns.2016.10.001. 28341039 10.1016/j.pjnns.2016.10.001

[45] Zong W, Lu X, Dong G, Zhang L, Li K. Molecular mechanisms of exercise intervention in alleviating the symptoms of autism spectrum disorder: targeting the structural alterations of synapse. Front Psychiatry. 2023;14. 10.3389/fpsyt.2023.1096503. 10.3389/fpsyt.2023.1096503 PMC10102432 37065903

[46] Mazzone L, Dooling SW, Volpe E, et al. Precision microbial intervention improves social behavior but not autism severity: A pilot double-blind randomized placebo-controlled trial[J]. Cell Host Microbe. 2024;32(1):106–e1166. 38113884 10.1016/j.chom.2023.11.021

[47] Chernikova MA, Flores GD, Kilroy E, Labus JS, Mayer EA, Aziz-Zadeh L. The Brain-Gut-Microbiome System: Pathways and Implications for Autism Spectrum Disorder. Nutrients. 2021;13(12):4497. 10.3390/nu13124497. 10.3390/nu13124497 PMC8704412 34960049

[48] O’callaghan J, O’toole PW. Lactobacillus: host-microbe relationships[J]. Curr Top Microbiol Immunol. 2013;358:119–54. 22102141 10.1007/82_2011_187

[49] Peng Y, Ma Y, Luo Z, et al. Lactobacillus reuteri in digestive system diseases: focus on clinical trials and mechanisms[J]. Front Cell Infect Microbiol. 2023;13:1254198. 37662007 10.3389/fcimb.2023.1254198 PMC10471993

[50] Janssen AWF, Katiraei S, Bartosinska B, et al. Loss of angiopoietin-like 4 (ANGPTL4) in mice with diet-induced obesity uncouples visceral obesity from glucose intolerance partly via the gut microbiota[J]. Diabetologia. 2018;61(6):1447–58. 29502266 10.1007/s00125-018-4583-5 PMC6449003

[51] Zheng Z, Lyu W, Ren Y, et al. Allobaculum involves in the modulation of intestinal ANGPTLT4 expression in mice treated by High-Fat Diet[J]. Front Nutr. 2021;8:690138. 34095196 10.3389/fnut.2021.690138 PMC8171929

[52] Pujo J, Petitfils C, Le Faouder P, et al. Bacteria-derived long chain fatty acid exhibits anti-inflammatory properties in colitis[J]. Gut. 2021;70(6):1088–97. 32978245 10.1136/gutjnl-2020-321173

[53] Nay K, Smiles WJ, Kaiser J, McAloon LM, Loh K, Galic S, et al. Molecular mechanisms underlying the beneficial effects of exercise on brain function and neurological disorders. Int J Mol Sci. 2021;22:4052. 10.3390/ijms22084052. 33919972 10.3390/ijms22084052 PMC8070923

[54] Barton W, Penney NC, Cronin O, et al. The Microbiome of professional athletes differs from that of more sedentary subjects in composition and particularly at the functional metabolic level[J]. Gut. 2018;67(4):625–33. 28360096 10.1136/gutjnl-2016-313627

[55] Matsumoto M, Inoue R, Tsukahara T, et al. Voluntary running exercise alters microbiota composition and increases n-butyrate concentration in the rat cecum[J]. Biosci Biotechnol Biochem. 2008;72(2):572–6. 18256465 10.1271/bbb.70474

[56] Erlandson KM, Liu J, Johnson R, et al. An exercise intervention alters stool microbiota and metabolites among older, sedentary adults[J]. Ther Adv Infect Dis. 2021;8:20499361211027067. 34262758 10.1177/20499361211027067 PMC8246564

[57] Han S, Tai C, Westenbroek RE, et al. Autistic-like behaviour in Scn1a+/- mice and rescue by enhanced GABA-mediated neurotransmission[J]. Nature. 2012;489(7416):385–90. 22914087 10.1038/nature11356 PMC3448848

[58] Han S, Tai C, Jones CJ, et al. Enhancement of inhibitory neurotransmission by GABAA receptors having alpha2,3-subunits ameliorates behavioral deficits in a mouse model of autism[J]. Neuron. 2014;81(6):1282–9. 24656250 10.1016/j.neuron.2014.01.016 PMC4079471

[59] Al-Otaish H, Al-Ayadhi L, Bjorklund G, et al. Relationship between absolute and relative ratios of glutamate, glutamine and GABA and severity of autism spectrum disorder[J]. Metab Brain Dis. 2018;33(3):843–54. 29397522 10.1007/s11011-018-0186-6

[60] Wan Y, Zuo T, Xu Z, Zhang F, Zhan H, Chan D, Ng SC. Underdevelopment of the gut microbiota and bacteria species as non-invasive markers of prediction in children with autism spectrum disorder. Gut. 2022;71(5):910-18. 10.1136/gutjnl-2020-324015 34312160

[61] Cavaliere G, Catapano A, Trinchese G, Cimmino F, Penna E, Pizzella A, et al. Butyrate improves neuroinflammation and mitochondrial impairment in cerebral cortex and synaptic fraction in an animal model of diet-induced obesity. Antioxidants. 2022;12:4. 10.3390/antiox12010004. 36670866 10.3390/antiox12010004 PMC9854835

[62] Singh V, Lee G, Son H, Koh H, Kim ES, Unno T, et al. Butyrate producers, the Sentinel of gut: their intestinal significance with and beyond butyrate, and prospective use as microbial therapeutics. Front Microbiol. 2023;13:1103836. 10.3389/fmicb.2022.1103836. 36713166 10.3389/fmicb.2022.1103836 PMC9877435

[63] Belén Sanz-Martos A, Fernández-Felipe J, Merino B, Cano V, Ruiz-Gayo M, Del Olmo N. Butyric acid precursor tributyrin modulates hippocampal synaptic plasticity and prevents Spatial memory deficits: role of PPARγ and AMPK. Int J Neuropsychopharmacol. 2022;25:498–511. 10.1093/ijnp/pyac015. 35152284 10.1093/ijnp/pyac015 PMC9211015

[64] Mansuy-Aubert V, Ravussin Y. Short chain fatty acids: the messengers from down below. Front Neurosci. 2023;17. 10.3389/fnins.2023.1197759. 10.3389/fnins.2023.1197759 PMC10359501 37483350

[65] Faraji N, Payami B, Ebadpour N, Gorji A. Vagus nerve stimulation and gut microbiota interactions: a novel therapeutic avenue for neuropsychiatric disorders. Neurosci Biobehav Rev. 2025;169:105990. 10.1016/j.neubiorev.2024.105990. 39716559 10.1016/j.neubiorev.2024.105990

[66] Launay J-M, Delorme R, Pagan C, Callebert J, Leboyer M, Vodovar N. Impact of IDO activation and alterations in the kynurenine pathway on hyperserotonemia, NAD + production, and AhR activation in autism spectrum disorder. Transl Psychiatry. 2023;13:380. 10.1038/s41398-023-02687-w. 38071324 10.1038/s41398-023-02687-w PMC10710433

[67] Wang F, Zhou H, Deng L, Wang L, Chen J, Zhou X. Serine deficiency exacerbates inflammation and oxidative stress via microbiota-gut‐brain axis in D‐galactose‐induced aging mice. Mediat Inflamm. 2020;20201:5821428. 10.1155/2020/5821428 PMC7071807 32189994

[68] Kristensen NB, Bryrup T, Allin KH, et al. Alterations in fecal microbiota composition by probiotic supplementation in healthy adults: a systematic review of randomized controlled trials[J]. Genome Med. 2016;8(1):52. 27159972 10.1186/s13073-016-0300-5 PMC4862129

[69] Maria Oliva-Hemker, Stacy A. Kahn, William J. Steinbach, SECTION ON GASTROENTEROLOY, HEPATOLOGY, AND NUTRITION, COMMITTEE ON INFECTIOUS DISEASES; Fecal Microbiota Transplantation: Information for the Pediatrician. Pediatrics December. 2023;152(6):e2023062922. 10.1542/peds.2023-062922. 10.1542/peds.2023-062922 37981872

[70] Kwak MJ, Kim SH, Kim HH, et al. Psychobiotics and fecal microbial transplantation for autism and attention-deficit/hyperactivity disorder: Microbiome modulation and therapeutic mechanisms[J]. Front Cell Infect Microbiol. 2023;13:1238005. 37554355 10.3389/fcimb.2023.1238005 PMC10405178

[71] Li N, Chen H, Cheng Y, et al. Fecal microbiota transplantation relieves Gastrointestinal and autism symptoms by improving the gut microbiota in an Open-Label Study[J]. Front Cell Infect Microbiol. 2021;11:759435. 34737978 10.3389/fcimb.2021.759435 PMC8560686

[72] Kang DW, Adams JB, Gregory AC, et al. Microbiota transfer therapy alters gut ecosystem and improves Gastrointestinal and autism symptoms: an open-label study[J]. Microbiome. 2017;5(1):10. 28122648 10.1186/s40168-016-0225-7 PMC5264285

[73] Chen K, Fu Y, Wang Y, et al. Therapeutic effects of the in vitro cultured human gut microbiota as transplants on altering gut microbiota and improving symptoms associated with autism spectrum Disorder[J]. Microb Ecol. 2020;80(2):475–86. 32100127 10.1007/s00248-020-01494-w

[74] Chen Y, Xueying Z, Jiaqu C, Qiyi C, Huanlong Q, Ning L, et al. FTACMT study protocol: a multicentre, double-blind, randomised, placebo-controlled trial of faecal microbiota transplantation for autism spectrum disorder. BMJ Open. 2022;12:e051613. 10.1136/bmjopen-2021-051613. 35105621 10.1136/bmjopen-2021-051613 PMC8804636

[75] Zhu B, Liang L, Huang Y, Wang H, Zhou J, Xiong D, et al. Exploring the relationship between the gut microbiota and cognitive function in schizophrenia patients with distinct weights. Schizophr Res. 2025;280:103–13. 10.1016/j.schres.2025.04.017. 40279867 10.1016/j.schres.2025.04.017

[76] Zhu B, Liang L, Chen S, Li H, Huang Y, Wang W, et al. Multi-kingdom microbial changes and their associations with the clinical characteristics in schizophrenia patients. Transl Psychiatry. 2025;15. 10.1038/s41398-025-03449-6. 10.1038/s41398-025-03449-6 PMC12228720 40617811

