# Supplementary material for: Gut microbiota mediates the beneficial effects of exercise on autism-like behaviors
Source: BMC Microbiol. 2026 Jan 12;26:157. doi: 10.1186/s12866-025-04632-x (PMC12927222; doi:10.1186/s12866-025-04632-x)
Supplement: Supplementary file 1 — Supplementary Material 1. [file 12866_2025_4632_MOESM1_ESM.docx]

**Supplementary File**

**Animal Experiments and Group Allocation**

To minimize litter effects and ensure the reliability of the experimental design, all VPA-exposed offspring used in this study were derived from at least four independent litters. No more than two pups from the same litter were included within a single experimental group, thereby avoiding pseudo-replication arising from treating same-litter individuals as independent samples. After early postnatal screening to confirm successful VPA modeling (e.g., absence of the neonatal “tail reflex”), all pups were sequentially numbered. A computer-generated randomization procedure stratified by litter of origin was then applied to assign animals to the exercise intervention group (E_ASD), the non-exercise ASD group, the fecal microbiota transplantation group (FMT), or the sham-transplantation group (sFMT). This stratified randomization ensured that each experimental group consisted of animals originating from multiple distinct litters, effectively reducing systematic bias associated with litter-specific variation. To further limit potential experimenter bias, all behavioral assessments, sample processing procedures, 16S rRNA sequencing, and quantification of short-chain fatty acids (SCFAs) and neurotransmitters were performed under blinded conditions, with investigators unaware of group identities throughout data collection and analysis.

**16S rRNA Gene Sequencing Analysis**

Fecal samples (100 mg each) collected from rats at postnatal day 23 (PND 23) were immediately flash-frozen in liquid nitrogen and stored at -80°C until processing. Genomic DNA was extracted using the MagPure Soil DNA LQ Kit (Magen Biotech, China). DNA concentration and purity were evaluated using a NanoDrop ND-2000 spectrophotometer (Thermo Fisher Scientific, USA) and confirmed by 1.2% agarose gel electrophoresis. The hypervariable V3–V4 regions of bacterial 16S rRNA genes were amplified using the universal primers 338F (5′-ACTCCTACGGGAGGCAGCA-3′) and 806R (5′-GGACTACHVGGGTWTCTAAT-3′). PCR amplification was performed in triplicate, with sterile water serving as a negative control. Amplicons were purified using Vazyme VAHTS™ DNA Clean Beads (N411-01, China) and quantified using the Quant-iT PicoGreen dsDNA Assay Kit (Invitrogen, USA) on a BioTek FLx800 microplate reader. Libraries were prepared using the Illumina TruSeq Nano DNA LT Library Prep Kit, followed by size selection through 2% agarose gel electrophoresis. The quality of the final libraries was validated using an Agilent 2100 Bioanalyzer with the High Sensitivity DNA Kit (Agilent Technologies, USA) and quantified using the Promega QuantiFluor fluorescence system. Paired-end sequencing (PE300) was performed on an Illumina NovaSeq 6000 platform, as provided by the sequencing service (Novogene, China).

**SCFA Profiling**

The seven SCFAs measured in this study were selected based on extensive ASD literature, as these metabolites play key roles in gut–brain signaling, immune modulation, and behavior. They also represent the core SCFAs that our targeted GC–MS/MS platform can reliably and quantitatively detect. Fecal samples (20 mg) collected at PND 23 were homogenized in 1 mL of 0.5% (v/v) phosphoric acid using a steel bead and tissue lyser (30 Hz, 3 cycles of 10 s). Following vortexing for 10 min and ice-bath sonication for 5 min, 100 μL of the supernatant was centrifuged (12,000 ×g, 10 min, 4°C). The resulting supernatant was mixed with 500 μL methyl tert-butyl ether (MTBE) containing internal standards, vortexed for 3 min, sonicated for 5 min, and centrifuged (12,000 ×g, 10 min, 4°C). The organic phase was collected and stored at -20°C until analysis. SCFAs were quantified using an Agilent 7890B gas chromatograph coupled with a 7000D triple quadrupole mass spectrometer (GC-MS/MS), equipped with a DB-5MS capillary column (30 m × 0.25 mm × 0.25 μm; Agilent J&W, USA). Helium was used as the carrier gas at a flow rate of 1.2 mL/min under splitless mode, with a 2 μL injection volume. The GC temperature program was set from 90°C to 230°C with controlled ramping and hold steps optimized for SCFA separation. Ion source and transfer line temperatures were set to 200°C and 230°C, respectively. Data acquisition was performed in multiple reaction monitoring mode. Quantification of SCFAs was performed using isotope-labeled internal standards (acetic acid-d4, propionic acid-d5, butyric acid-d7; Sigma-Aldrich) added prior to extraction. Calibration curves were constructed using seven concentration levels covering the expected physiological range (0.5–500 μM), with all analytes showing excellent linearity (R² ≥ 0.995). Limits of detection (LOD) and limits of quantification (LOQ) were calculated at signal-to-noise ratios of 3 and 10, respectively, and ranged from 0.05–0.20 μM (LOD) and 0.10–0.50 μM (LOQ). Method recovery, assessed by spiking pooled fecal matrices at low, medium, and high levels, ranged between 85–112%. Intra- and inter-batch precision (RSD) were <10% and <15%, respectively, demonstrating good analytical stability throughout the batch runs.

**Neurotransmitter Quantification**

Likewise, the 55 neurotransmitters and their related precursors/metabolites were selected for their well-established biological relevance to ASD and their involvement in key neurochemical pathways linked to emotional regulation and gut–brain communication. Prefrontal cortex tissues (1 mm anterior to bregma) collected at PND 28 were homogenized in 500 μL of ice-cold 70% methanol using a tissue lyser (30 Hz, 4 cycles of 30 s). After centrifugation (12,000 ×g, 10 min, 4°C), 100 μL of the supernatant was analyzed by LC-MS/MS. Chromatographic separation was achieved using a Waters ACQUITY UPLC HSS T3 C18 column (100 × 2.1 mm, 1.8 μm), with a mobile phase consisting of 0.1% formic acid in water (A) and 0.1% formic acid in acetonitrile (B). The gradient elution was as follows: 5% B (0 min) → 95% B (8 min) → 95% B (9.5 min) → 5% B (12 min) at a flow rate of 0.35 mL/min. The column temperature was maintained at 40°C, with an injection volume of 2 μL. For neurotransmitter analysis, a panel of isotope-labeled internal standards (e.g., serotonin-d4, dopamine-d4, GABA-d6; Cayman Chemical) was included to correct for extraction and ionization variability. Calibration curves covering 0.1–1000 ng/mL were constructed for all analytes, yielding linearity values of R² ≥ 0.990. LOD and LOQ values ranged from 0.01–0.10 ng/mL and 0.05–0.50 ng/mL, respectively. Method recovery assessed from tissue-matrix spike-and-recovery experiments was between 80–115%. Intra-batch and inter-batch variation remained within acceptable analytical limits (RSD <12% and <18%, respectively).

**Behavioral Assessments**

Rats underwent behavioral testing, including the Open Field Test and the Three-Chamber Social Interaction Test. In the Open Field Test, rats were individually placed in the center of a plexiglass arena (50 × 50 × 50 cm³) and allowed to explore freely for 5 min. The total distance traveled, time spent in the central zone (25% of the total area), and frequency of center entries were recorded using EthoVision XT 15 software (Noldus, Netherlands). The arena was sanitized with 75% ethanol between trials. For the Three-Chamber Social Interaction Test, rats were acclimated to the testing room for 24 h prior to the experiments. The apparatus consisted of three interconnected chambers (50 × 50 × 50 cm³ each). In Phase 1 (habituation), rats explored two empty wire cages placed in the side chambers for 5 min. In Phase 2 (social novelty), a novel conspecific (Stranger 1, S1) was placed in the left chamber, and exploration time was recorded for 10 min. In Phase 3 (social preference), a second novel rat (Stranger 2, S2) was placed in the right chamber, and interaction times with S1 vs. S2 were analyzed over 10 min. All sessions were video-recorded, and the chambers were cleaned with 70% ethanol between trials.


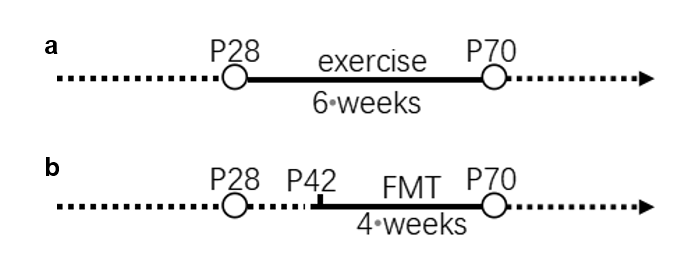


**Fig. S1. Schematic illustration of the experimental timeline.**

(a) Exercise intervention: Rats underwent a 6-week treadmill training protocol starting at P28, with behavioral testing and sample collection performed at P70. (b) FMT protocol: ASD models were induced at P28, and from P42 rats received a 4-week fecal microbiota transplantation from exercise-treated ASD donors, followed by behavioral assessment and tissue collection at P70.


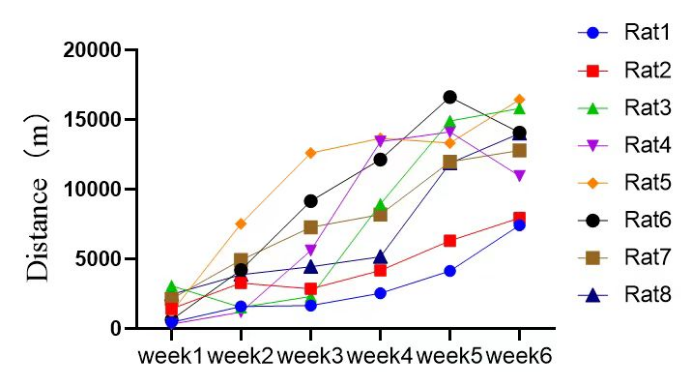


**Fig. S2. Individual running distance trajectories during the 6-week treadmill training period.**

Rats progressively increased their running distance and reached a stable level over time, indicating good adherence to the exercise protocol.


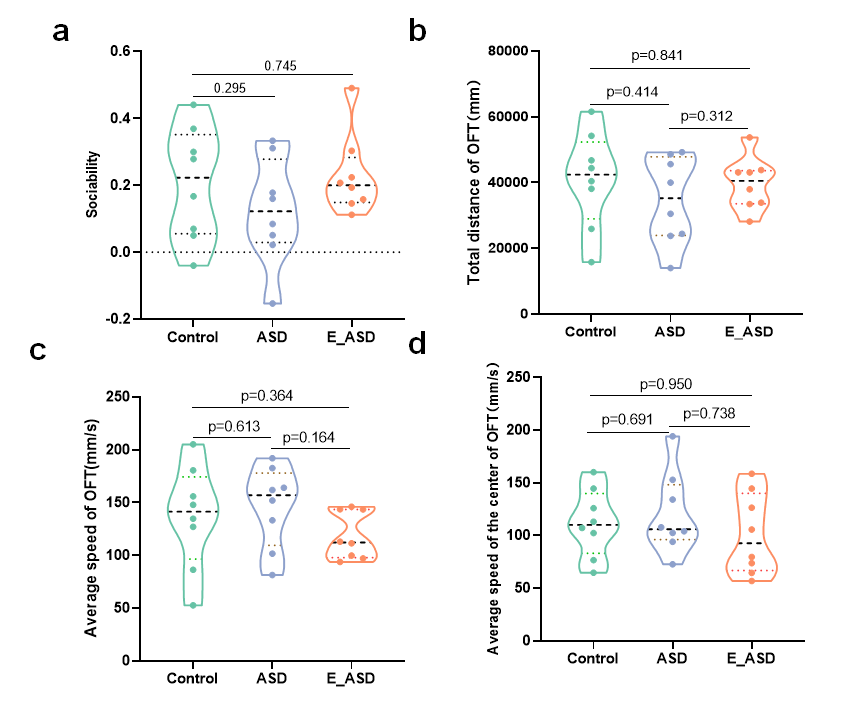


**Fig. S3. Effects of exercise on social behavior and OFT measures in ASD rats.**

No significant group differences were observed in social behavior or OFT parameters for the E_ASD group.


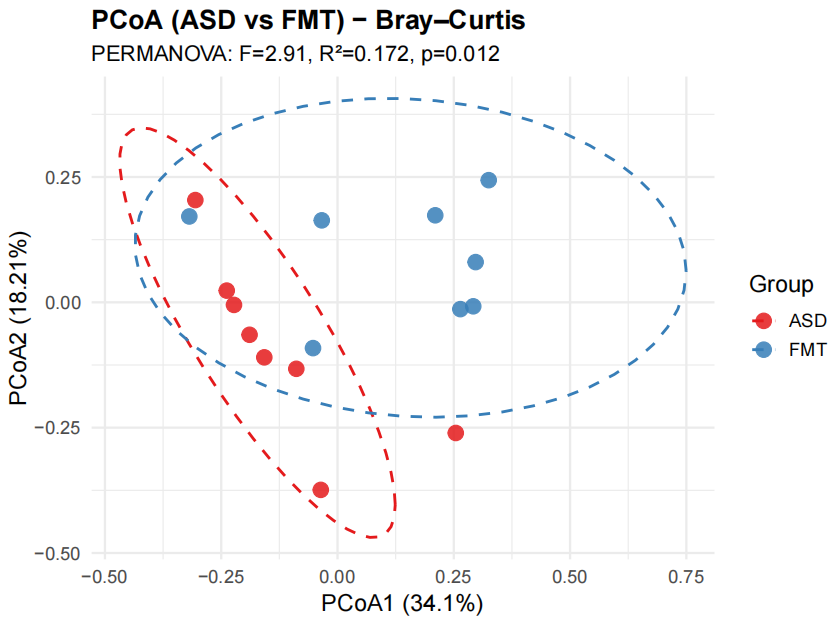


**Fig. S4. Bray-Curtis PCoA of gut microbiota after FMT.**

Clear separation was observed between the FMT and ASD groups, indicating that exercise-derived microbiota reshaped the recipient microbial community.


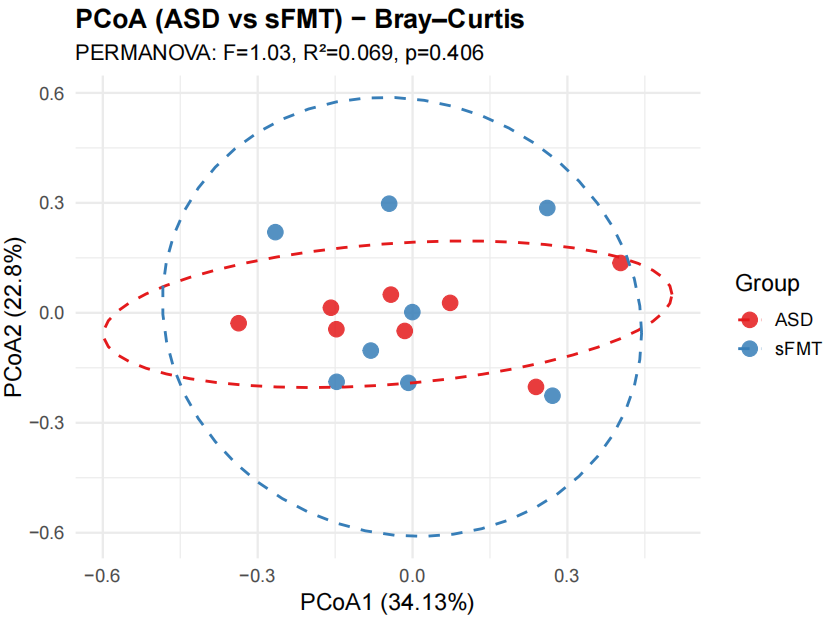


**Fig. S5. Bray-Curtis PCoA of gut microbiota after sFMT.**

No significant separation was observed between the sFMT and ASD groups, suggesting that saline gavage did not alter the recipient gut microbiota.
